# Enhancing Neuromuscular Conditioning in Football Players Through Single‐Leg and Double‐Leg Cycling: A Randomized Controlled Trial

**DOI:** 10.1155/tsm2/5535929

**Published:** 2026-02-27

**Authors:** Jitin Chahal, Moattar Raza Rizvi, Ankita Sharma, Shishir Nigam, Waqas Sami

**Affiliations:** ^1^ Department of Physiotherapy, School of Allied Health Sciences, Manav Rachna International Institute of Research & Studies, (MRIIRS), Faridabad, 121004, India; ^2^ Faculty of Allied Health Science, Santosh Deemed to be University, Santosh Nagar, Ghaziabad, 201009, Delhi NCR, India; ^3^ Department of Physiotherapy, Amity Institute of Health Allied Sciences, Amity University, Noida, 201313, Uttar Pradesh, India, amity.edu; ^4^ School of Physiotherapy and Rehabilitation Sciences, K. R. Mangalam University, Gurugram, 122003, Haryana, India, krmangalam.edu.in; ^5^ Department of Pre-Clinical Affairs, College of Nursing, QU Health, Qatar University, P.O. Box 2713, Doha, Qatar, qu.edu.qa

**Keywords:** agility, anaerobic power, football conditioning, isokinetic strength, knee flexor strength, single-leg cycling, sprint performance

## Abstract

**Background:**

Football requires high levels of neuromuscular conditioning to meet the demands of explosive actions such as sprinting, cutting, and kicking, while minimizing the risk of lower limb injuries—especially hamstring strains linked to muscle imbalances. Single‐leg cycling (SLC), a form of unilateral training, may offer superior neuromuscular adaptations compared to traditional double‐leg cycling (DLC). This study aimed to compare the effects of SLC and DLC on lower limb strength, anaerobic power, fatigue resistance, agility, and sprint performance in competitive football players.

**Methods:**

A four‐week, assessor‐blinded randomized controlled trial was conducted on 42 male football players (aged 18–26), allocated equally to SLC (*n* = 21) and DLC (*n* = 21) groups. Both groups underwent biweekly cycling sessions. Pre‐ and postintervention assessments included isokinetic peak torque of knee flexors and extensors, hamstring‐to‐quadriceps (H/Q) ratio, peak and minimum power, fatigue index (RAST), 20‐m zig‐zag agility test, and 30‐m Sprint Test.

**Results:**

Both groups showed significant improvements in anaerobic performance metrics (peak and minimum power, *p* < 0.001). However, SLC yielded significantly higher gains in knee flexor peak torque (Δ + 46.7%, *p* < 0.001) and H/Q ratio (Δ + 30.2%, *p* = 0.02), indicating superior hamstring activation and improved muscle balance. Fatigue index significantly decreased only in the SLC group (Δ − 7.3%, *p* = 0.04), reflecting enhanced anaerobic endurance. SLC also resulted in greater improvements in agility (Δ − 5.9%) and sprint performance (Δ − 8.1%) compared to DLC (*p* < 0.001), suggesting improved neuromuscular coordination and explosive capability. Knee extensor torque increased modestly in both groups, with no significant between‐group difference (*p* = 0.46).

**Conclusion:**

SLC training offers superior benefits over DLC in improving lower limb strength balance, fatigue resistance, agility, and sprinting in football players. It may serve as a targeted conditioning strategy to enhance performance and reduce injury risk in sport‐specific contexts.

**Trial Registration:** Clinical Trials Registry–India (CTRI): CTRI/2023/06/053941

## 1. Introduction

Football performance depends on technical skills—dribbling, passing, shooting, and defending—alongside key physical attributes, including endurance, strength, agility, and coordination [1]. The sport’s high‐intensity, intermittent nature places substantial demands on the lower limbs, especially the quadriceps and hamstrings [2]. Rapid sprints, directional changes, and kicking can lead to overuse or acute injuries. Longitudinal data highlight hamstring injuries as a recurrent issue in professional football, with muscle strength imbalances increasing injury risk [3, 4]. Targeted conditioning programs that improve lower limb strength and neuromuscular control are therefore vital to reduce injury incidence and enhance performance [5].

Physiological parameters such as knee extensor and flexor strength strongly correlate with football‐specific actions, including kicking proficiency [6]. Researchers increasingly emphasize exercises geared toward higher angular velocities, given the speed and explosive nature of competitive matches [7, 8]. Evidence also suggests that well‐structured strength and power training can boost sprint performance and agility, both of which are critical to success on the pitch [9]. To translate laboratory findings into practice, coaches and sports scientists continuously search for innovative training strategies that simultaneously address performance goals and injury prevention [10].

Cycling is increasingly recognized as a low‐impact method for improving aerobic capacity and muscular endurance while minimizing joint stress [11]. Athletes often integrate cycling sessions to assist in recovery or augment cardiovascular fitness. Single‐leg cycling (SLC), in particular, represents a specialized form of unilateral training, intended to improve strength, balance, power, and neuromuscular coordination in each limb independently [12]. Pedaling with only one leg places a relatively higher load on the active muscles than bilateral cycling, potentially eliciting more pronounced adaptations in targeted muscle groups [13]. Furthermore, SLC appears to induce greater cardiovascular strain—evidenced by elevated heart rate (HR) and ventilation—compared to equivalent two‐leg intensities [14, 15]. Improvements in muscular endurance have also been noted in both the trained and untrained limbs, reinforcing SLC’s relevance for strengthening unilateral movement patterns [11].

To quantify the effectiveness of such interventions, isokinetic dynamometry remains a reliable tool for measuring peak torque, total work, and strength ratios under controlled conditions [16]. This method is well established in football research, where knee extensor and flexor assessments help delineate performance capabilities and injury risks [17, 18]. Velocity‐specific measures are particularly informative for sports like football, where repeated, high‐speed movements are routine. Latham, for instance, reported significant gains in knee strength at 500°/s and an improved hamstring‐to‐quadriceps (H/Q) ratio in players over a competitive season, illustrating how high‐velocity strength work aligns with on‐field demands [19].

The link between lower limb strength and football performance extends beyond injury mitigation to include critical outcomes such as sprinting speed, explosive power, and agility [9]. Notably, the risk of hamstring strain is closely tied to deficits in eccentric hamstring capacity and imbalances with quadriceps strength [3, 5]. Although various eccentric‐focused exercises have proven beneficial—such as Nordic hamstring curls [10]—the potential for SLC to target unilateral deficits and enhance high‐speed strength remains underexplored. Given football’s frequent one‐leg propulsion (e.g., striking the ball, single‐leg landing, cutting maneuvers), SLC may offer additional, sport‐specific benefits [20].

Accordingly, this study aims to compare single‐leg versus conventional double‐leg cycling (DLC) workouts in football players aged 18–26 years to determine their effects on maximum peak torque and peak torque ratios. We hypothesize that single‐leg high‐intensity cycling training will elicit significantly greater improvements in lower limb strength parameters, providing a novel training avenue to optimize performance and reduce injury risk.

By examining these adaptations, our findings may help coaches, trainers, and sports scientists refine strength and conditioning programs. Incorporating SLC could prove especially valuable in augmenting traditional football training protocols, addressing unilateral deficits and high‐velocity demands inherent to the sport. Ultimately, such targeted interventions may translate into stronger, more agile athletes who are better equipped to handle the physical and tactical requirements of competitive match play.

## 2. Methodology

### 2.1. Study Design

This investigation was structured as a 4‐week, assessor‐blinded, randomized controlled trial (RCT) in adherence to the Consolidated Standards of Reporting Trials (CONSORT) guidelines. Participants were assessed before and after the intervention period to identify any changes resulting from single‐leg cycling (SLC) or Double‐leg cycling (DLC) programs. DLC served as an active comparator, consistent with exercise‐intervention RCT methodology. All procedures took place at the Manav Rachna Physiotherapy Outpatient Department and the Manav Rachna Sports Science Centre, ensuring a consistent and controlled setting for training and follow‐up measurements.

### 2.2. Sample Size Calculation

The sample size estimation for this study was conducted using G∗Power software (v3.1.9.4, Heinrich Heine University, Düsseldorf, Germany) to ensure adequate statistical power for detecting meaningful differences in the primary outcome between groups. An a priori power analysis for a repeated‐measures ANOVA (within‐between interaction) was performed. Assuming a medium effect size (*f* = 0.25), a power (1 − β) of 0.80, a correlation among repeated measures of 0.5, and a nonsphericity correction (*ε*) of 1, the required total sample size was 34 participants for two groups measured at two time points (pre and post). These parameters were selected in accordance with standard recommendations for clinical and sports science research to minimize the risk of type I and type II errors. To account for an anticipated 20% dropout rate, the final target sample size was adjusted to 41 participants. However, to ensure equal distribution across the two study groups, the sample was rounded up to 42 participants, with 21 allocated to each group. Sixty football players were screened to recruit a sample that met the inclusion criteria.

### 2.3. Participant

**FIGURE 1:**
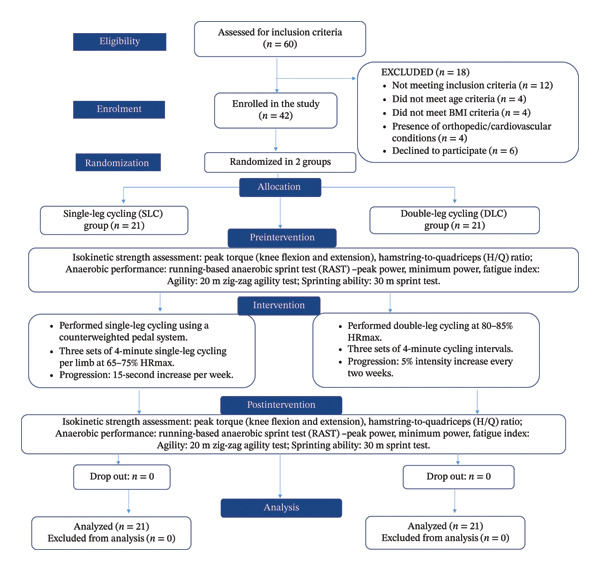
Consort chart of the study design summarizing participant flow, interventions, and outcome measures.

Sixty male football players were initially screened via purposive sampling (Figure 1). Eligibility required that participants be 18–26 years old, compete at or above district level, have practiced football for at least six months (three to four sessions weekly), and hold a body mass index (BMI) of 18.5–24.9. Exclusion criteria included resting systolic blood pressure above 90 mmHg or diastolic blood pressure above 140 mmHg, any neurological, orthopedic, or cardiovascular conditions, a history of blood clotting disorders, use of medications or dietary supplements, and smoking within the past 6 months. Forty two players met these conditions, provided written informed consent, and were fully briefed on all risks, benefits, and the option to withdraw from the study at any time.

### 2.4. Ethical Approval

Ethical clearance was obtained from the Department of Physiotherapy’s ethical committee at the Allied Health Science Faculty, Manav Rachna International Institute of Research and Studies (approval no. MRIIRS/FAHS/PT/2022‐23/S‐009). The researcher covered all investigation‐related expenses, ensuring no financial burden on the participants.

### 2.5. Randomization and Allocation

A computer‐generated random allocation sequence assigned participants in a 1:1 ratio to either SLC or DLC. Allocation was concealed using sealed, opaque envelopes, opened only after baseline data collection. Each group contained 21 participants. The trial was assessor‐blinded; while participants were aware of their assigned cycling protocol, the outcome assessor remained blinded to group allocation throughout the intervention and testing phases.

### 2.6. Familiarization Session

Before starting the main intervention, participants attended a supervised familiarization session to ensure proper technique and comfort with the equipment. They were introduced to the electromagnetically braked cycle ergometer, where they performed short submaximal pedaling intervals (either single‐ or double‐leg) under close supervision. Likewise, they received guidance on the isokinetic dynamometer procedure, practicing a few submaximal knee flexion and extension trials.

### 2.7. Interventions

The SLC and DLC protocols were implemented through a structured cycling training program adapted from previous study [21]. The intervention was conducted over a 4‐week period, with participants completing two training sessions per week on the same model of electromagnetically braked cycle ergometer (Concept2 BikeErg, India). Each session incorporated a standardized warm‐up, structured intervention phase (SLC and DLC) and a cool‐down period. HR and rating of perceived exertion (RPE) were continuously monitored to regulate training intensity and mitigate excessive physiological strain.

Each session commenced with a 10‐min warm‐up, consisting of 5 minutes of low‐intensity DLC at 50%–60% of HRmax, followed by 5 minutes of dynamic stretching targeting the quadriceps, hamstrings, and hip flexors. HRmax was recorded at the conclusion of the warm‐up to establish a baseline cardiovascular response before initiating the main intervention. The SLC protocol was implemented using a counterweighted pedal system to maintain biomechanical efficiency. Participants completed three sets of 4‐minute SLC intervals per limb, maintaining an intensity of 65%–75% of HRmax. A 6‐minute active recovery period at 40% HRmax was incorporated between intervals. Training progression was achieved by increasing the duration of the single‐leg intervals by 15 s per week, ensuring HR remained within the prescribed range.

The DLC protocol consisted of three sets of 4‐minute high‐intensity cycling intervals, performed at 80%–85% of HRmax, interspersed with 6‐minute active recovery phases at 50% HRmax. Progressive overload was applied by increasing the intensity by 5% every 2 weeks to optimize endurance adaptations. HR was monitored continuously, while RPE was recorded postinterval to assess subjective exertion. If participants exhibited an RPE exceeding 17 (“very hard”) [22] or HR values surpassing 90% of HRmax, the training load was immediately adjusted to prevent excessive fatigue and cardiovascular strain.

Following the intervention phase, each session concluded with a 5‐minute cool‐down, consisting of low‐intensity cycling at 40%–50% HRmax, followed by 5 minutes of static stretching targeting the lower limb musculature to facilitate recovery and minimize postexercise stiffness. Given the variability in anthropometric and biomechanical characteristics among football players, modifications to cycling ergonomics were permitted based on individual requirements. Adjustments in saddle height, handlebar reach, and pedal positioning were made as necessary to optimize biomechanical efficiency, power transfer, and lower limb joint alignment, thereby reducing the risk of musculoskeletal strain.

Both the groups were instructed to continue their regular football training routine throughout the intervention periods. The regular training was not modified or standardized as the aim of the study was to evaluate the effects of the cycling interventions under real‐world training conditions. Participants were instructed to avoid any additional structured lower limb strength and conditioning programs during the study period.

Throughout the intervention period, participants reported mild quadriceps fatigue and delayed‐onset muscle soreness (DOMS), particularly following SLC sessions. Symptoms were transient and resolved within 48 h, without interfering with subsequent training sessions. Two participants reported minor knee discomfort, necessitating saddle height adjustments to optimize knee kinematics and pedaling efficiency. No severe adverse events, including cardiovascular distress or excessive musculoskeletal strain, were recorded. If HR exceeded 90% of HRmax, workload intensity was immediately reduced to prevent excessive cardiovascular stress. Adequate hydration was maintained throughout all sessions to prevent dehydration‐related complications. The protocol was structured to allow for individualized modifications, while ensuring standardized intensity levels, thereby optimizing physiological adaptations and minimizing injury risk among football players integrating cycling into their training regimens. Nutritional guidelines were provided to facilitate recovery and muscle adaptation, particularly following high‐intensity cycling sessions. Participants were encouraged to consume protein‐rich meals post‐training to support muscle repair and glycogen replenishment. Individualized dietary recommendations were given based on body composition assessments to ensure adequate caloric intake for training demands.

### 2.8. Outcome Measures

#### 2.8.1. Running‐Based Anaerobic Sprint Test (RAST)

The RAST was conducted following a previously established protocol [23–25] to assess anaerobic performance parameters, including peak power, average power, minimum power, and fatigue index [23]. The test was performed on a 400‐m standard running track, where six 35‐m sprints were conducted with a 10‐s recovery period between each sprint. The sprint course was marked using cones placed at 0 and 35 m, ensuring a standardized distance for all trials. Participants were instructed to sprint maximally from the start to the finish line for each repetition. A trained timekeeper recorded the sprint time using a handheld stopwatch for each sprint attempt.

The power output for each sprint was calculated using the equation Power = (Weight × Distance^2^)/Time^3^, which provided individual values for peak power (highest recorded power output), minimum power (lowest recorded power output), and average power (mean power across all sprints). Additionally, the fatigue index, which represents the rate of decline in power across the six sprints, was determined using the formula (Maximum Power − Minimum Power)/Total Sprint Time. This test setup allowed for the objective measurement of anaerobic capacity and fatigue resistance, both of which are critical for high‐intensity sports such as football.

#### 2.8.2. Isokinetic Strength Assessment

All isokinetic strength assessments were conducted at baseline and postintervention by a blinded assessor using an isokinetic dynamometer to evaluate concentric knee extension and flexion strength of the dominant leg at angular velocities of 30°/s, 60°/s, and 180°/s [26, 27]. This method was chosen due to its reliability and validity in assessing neuromuscular function and lower limb strength performance.

Participants were seated upright in the dynamometer chair with their trunk and thigh securely strapped to minimize extraneous body movements that could affect measurement accuracy. The lateral femoral epicondyle was precisely aligned with the axis of rotation of the dynamometer’s lever arm to ensure consistent torque measurement. The lower leg was securely attached to the lever arm at the lateral malleolus, ensuring proper positioning and stabilization throughout the testing procedure.

A standardized warm‐up protocol was administered before testing, consisting of three submaximal contractions followed by two maximal efforts, with 2‐minute rest intervals between trials to prevent early fatigue. After the warm‐up, participants performed five maximal voluntary contractions at each angular velocity for both knee extension and knee flexion. The highest peak torque value obtained across the trials was recorded for data analysis.

Additionally, the H/Q ratio was calculated by dividing the peak knee flexor torque by peak knee extensor torque at each angular velocity [28]. This ratio is a key indicator of muscle balance around the knee joint, providing insight into potential muscle imbalances, injury risk, and rehabilitation progress. A higher H/Q ratio is generally associated with better knee joint stability and lower injury susceptibility, particularly in high‐performance athletes.

#### 2.8.3. 20‐m Zig‐Zag Agility Test

The 20‐m zig‐zag agility test was conducted following a previously established protocol to assess agility performance, which is crucial for football players’ ability to rapidly change direction and accelerate during play [29]. The test was performed on a flat, nonslippery surface, where participants were required to sprint through a zig‐zag course as quickly as possible. The course was marked using five cones, positioned in a zig‐zag pattern over a 20‐m distance, with each cone placed at approximately 4‐m intervals. Participants were instructed to navigate around the cones using quick directional changes while maintaining maximum speed. A trained timekeeper recorded the sprint time using a handheld stopwatch for each attempt. Participants completed two trials, with a 1‐min recovery period between attempts. The fastest recorded time (shortest duration) was considered the final agility score.

#### 2.8.4. 30‐m Sprint Test

The 30‐m sprint test was conducted to evaluate sprint performance and acceleration ability in football players using previously described methodology [30]. The test was performed on a flat, nonslippery surface, such as artificial turf or an athletics track, with a 30‐m sprint lane marked using cones at the 0 m (start line) and 30 m (finish line). Players began in a standing position with one foot behind the starting line and were instructed to sprint at maximum effort upon a verbal cue. Sprint time was recorded using a handheld stopwatch, with the assistant starting the timer at the athlete’s first movement and stopping it when the torso crossed the finish line. Each participant completed two trials, with a 2‐min passive recovery period between sprints, and the fastest recorded time (in seconds) was used for analysis.

### 2.9. Statistical Analysis

All statistical analyses were performed using SPSS software (version 24, IBM Corp., Armonk, NY, USA). Descriptive statistics, including mean (M) and standard deviation (SD), were calculated for all outcome variables. The normality of data distribution was assessed using the Shapiro–Wilk test, and Levene’s test for equality of variances was conducted to ensure homogeneity of variance across groups. Baseline comparisons between the SLC group and the DLC group were performed using independent samples *t*‐tests for continuous variables and chi‐squared tests for categorical variables. Within‐group changes from pre‐ to postintervention were analyzed using paired *t*‐tests. The repeated measures analysis of variance (ANOVA) was used to detect within‐group differences over time, incorporating (pre vs. post), group (SLC vs. DLC), and their interaction effects. Effect sizes were calculated using Cohen’s d for *t*‐tests and partial eta squared (*η* ^2^ *p*) for ANOVA, with significance set at *p* < 0.05. Post hoc analyses were conducted using Bonferroni corrections to adjust for multiple comparisons, ensuring robust statistical inference.

## 3. Results

Baseline analysis confirmed that all variables followed a normal distribution, as assessed by the Shapiro–Wilk test. Independent sample *t*‐tests showed no statistically significant differences between the SLC and DLC groups across demographic and performance variables (all *p* > 0.05; Table 1). For instance, the mean age of participants was 20.90 ± 2.57 years in SLC and 21.76 ± 2.30 years in DLC (*p* = 0.26). Similarly, BMI was 21.39 ± 2.07 kg/m^2^ in the SLC group and 21.75 ± 2.05 kg/m^2^ in the DLC group (*p* = 0.58). Performance measures such as baseline peak torque for knee flexion (SLC: 60.48 ± 11.50 nm; DLC: 61.67 ± 10.17 nm, *p* = 0.72), agility time (SLC: 6.56 ± 0.09 s; DLC: 6.60 ± 0.10 s, *p* = 0.21), and 30‐m sprint time (SLC: 4.31 ± 0.06 s; DLC: 4.33 ± 0.05 s, *p* = 0.16) also showed no meaningful between‐group differences. These results confirm baseline homogeneity and allow for valid interpretation of intervention effects.

**TABLE 1:** Baseline comparison of participant characteristics and performance metrics using independent sample *t*‐test between single‐leg cycling (SLC) and double‐leg cycling (DLC) groups.

Variable	DLC (*n* = 21) (mean ± SD)	SLC (*n* = 21) (mean ± SD)	*t*	*p*
Age (years)	21.76 ± 2.30	20.90 ± 2.57	−1.14	0.26
Weight (kg)	68.24 ± 8.16	66.76 ± 8.18	0.59	0.56
Height (cm)	176.98 ± 4.53	176.50 ± 4.36	0.35	0.73
BMI (kg/m^2^)	21.75 ± 2.05	21.39 ± 2.07	0.56	0.58
Frame size (inches)	19.10 ± 1.18	18.67 ± 1.11	1.21	0.23
Saddle—pedal (inches)	25.19 ± 1.86	25.05 ± 2.40	0.22	0.83
Handlebar—saddle (inches)	21.62 ± 2.09	22.10 ± 4.22	−0.90	0.37
Peak power (W)	797.25 ± 161.72	849.71 ± 129.93	−1.16	0.25
Min power (W)	374.15 ± 47.36	377.28 ± 64.00	−0.18	0.86
Fatigue index (%)	51.67 ± 9.06	55.30 ± 6.10	−1.52	0.14
Peak torque flexion (nm)	61.67 ± 10.17	60.48 ± 11.50	0.36	0.72
Peak torque extension (nm)	138.33 ± 14.57	144.76 ± 28.26	−0.93	0.36
Ratio peak torque	0.45 ± 0.08	0.43 ± 0.10	0.73	0.47
Agility (sec)	6.60 ± 0.10	6.56 ± 0.09	1.28	0.21

Table 2 summarizes the effects of the intervention on all outcome variables, including time effects, group differences, and interaction terms derived from repeated measures ANOVA and post hoc comparisons. Following 4 weeks of training, both groups exhibited clear improvements in anaerobic performance, as assessed by the RAST. Peak power increased significantly in both the SLC (*p* < 0.001, $\eta_{p}^{2}$ = 0.49) and DLC (*p* < 0.001) groups, indicating effective neuromuscular adaptation. Similarly, minimum power improved over time in both conditions (*p* < 0.001, $\eta_{p}^{2}$ = 0.88), reflecting enhanced sustained output. However, only the SLC group demonstrated a significant reduction in fatigue index (*p* = 0.04, $\eta_{p}^{2}$ = 0.18), while the DLC group showed no change (*p* = 0.90). This suggests that unilateral cycling may offer superior benefits in mitigating fatigue across repeated sprint efforts.

**TABLE 2:** Repeated‐measures ANOVA and post hoc paired t‐test results comparing pre‐ and postintervention performance outcomes between the single‐leg cycling (SLC) and double‐leg cycling (DLC) groups.

Outcome measures	SLC		DLC		Repeated measure ANOVA			Post hoc within‐group comparison	
	Pre mean ± SD	Post mean ± SD	Pre mean ± SD	Post mean ± SD	Time *η* ^2^ *p* (p)	Group *η* ^2^ *p* (p)	Time × group *η* ^2^ *p* (p)	SLC pre vs post *t* (p)	DLC pre vs post *t* (p)
Peak power (W)	849.71 ± 129.93	940.80 ±0 .16	797.25 ± 161.72	884.57 ± 143.88	0.49 (< 0.001)	0.03 (0.24)	4.35 × 10^−4^ (0.90)	−4.50 (< 0.001)	−4.31 (< 0.001)
Minimum power (W)	377.28 ± 64.00	448.73 ± 70.84	374.15 ± 47.36	435.34 ± 54.64	0.88 (< 0.001)	5.18 × 10^−3^ (0.65)	0.04 (0.19)	−13.24 (< 0.001)	−11.34 (< 0.001)
Fatigue index (%)	55.30 ± 8.60	51.24 ± 9.33	51.67 ± 9.06	49.78 ± 8.69	0.18 (5.37 × 10^−3^)	0.03 (0.29)	0.03 (0.29)	2.84 (0.04)	1.32 (0.90)
Peak torque flexion (nm)	60.48 ± 11.50	88.71 ± 22.41	61.67 ± 10.17	67.10 ± 17.34	0.47 (< 0.001)	0.13 (0.02)	0.29 (< 0.001)	−7.12 (< 0.001)	−5.44 (< 0.001)
Peak torque extension (nm)	144.76 ± 28.26	158.10 ± 15.12	138.33 ± 14.57	144.76 ± 16.77	0.10 (0.04)	0.15 (0.01)	0.01 (0.46)	−2.02 (0.30)	−0.97 (0.90)
Ratio peak torque	0.43 ± 0.10	0.56 ± 0.15	0.45 ± 0.08	0.47 ± 0.14	0.23 (1.26 × 10^−3^)	0.04 (0.21)	0.13 (0.02)	−4.16 (< 0.001)	−3.17 (0.02)
Agility	6.56 ± 0.09	6.17 ± 0.10	6.60 ± 0.10	6.38 ± 0.10	0.84 (< 0.001)	0.45 (< 0.001)	0.29 (< 0.001)	13.12 (< 0.001)	14.26 (< 0.001)
30‐m sprint	4.31 ± 0.05	3.96 ± 0.07	4.33 ± 0.05	4.00 ± 0.05	0.97 (< 0.001)	0.11 (0.03)	0.02 (0.34)	24.96 (< 0.001)	23.60 (< 0.001)

*Note:* *η* ^2^ *p* interpretation—small (0.01), medium (0.06), and large (0.14). Post hoc: Bonferroni. Sphericity not a concern (two time points).

Strength‐related adaptations were also observed, particularly in the isokinetic assessment of the knee joint. Knee flexor peak torque increased significantly over time in both groups, with a more pronounced improvement in the SLC group, as indicated by a significant time × group interaction (*p* < 0.001, $\eta_{p}^{2}$ = 0.29). Although extensor torque also improved modestly (*p* = 0.04, $\eta_{p}^{2}$ = 0.10), no significant interaction was found (*p* = 0.46), suggesting that strength gains in this muscle group were not strongly dependent on the training modality. Importantly, the H/Q ratio improved more substantially in the SLC group (*p* < 0.001, $\eta_{p}^{2}$ = 0.23), with a significant interaction effect (*p* = 0.02, $\eta_{p}^{2}$ = 0.13), reflecting better muscle balance and potentially reduced injury risk.

Improvements in functional performance were evident in both agility and sprint tests. Agility times decreased significantly in both groups (*p* < 0.001, $\eta_{p}^{2}$ = 0.84), though the SLC group exhibited a larger effect (*p* < 0.001, $\eta_{p}^{2}$ = 0.45) and a significant interaction (*p* < 0.001, $\eta_{p}^{2}$ = 0.29), indicating greater responsiveness to unilateral training. Similarly, 30‐m sprint times improved in both groups (*p* < 0.001), with a modest group effect favoring SLC (*p* = 0.03, $\eta_{p}^{2}$ = 0.11) but no interaction (*p* = 0.34).

As depicted in Figure 2, participants in the SLC group demonstrated consistently greater pre–post‐improvements across key neuromuscular outcomes. Notably, the largest gains were observed in the knee flexor strength (*t* = −7.63, *p* < 0.001) and H/Q ratio (*t* = −3.90, *p* < 0.001), reinforcing the value of unilateral cycling in targeting muscle symmetry, functional power, and resilience to fatigue. While both training protocols yielded meaningful enhancements in anaerobic output and sprinting ability, the single‐leg approach appeared more effective in fostering task‐specific adaptations relevant to football performance.

FIGURE 2Paired *t*‐test comparisons of pre‐ and postintervention changes in performance variables for single‐leg cycling (SLC) and double‐leg cycling (DLC) groups.

**Figure:**
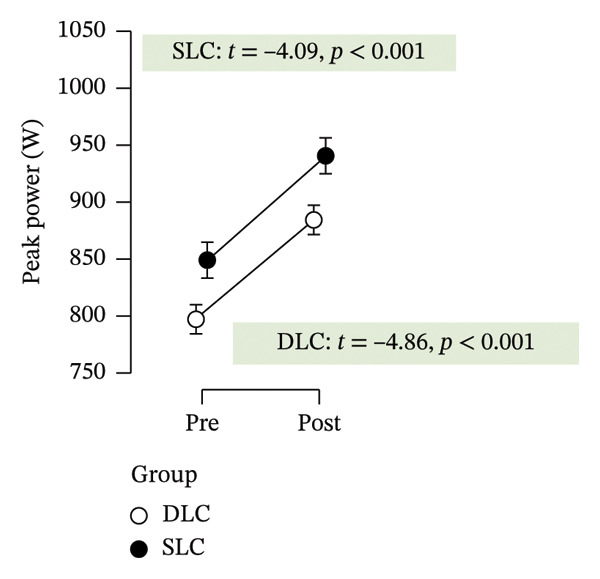
(a)

**Figure:**
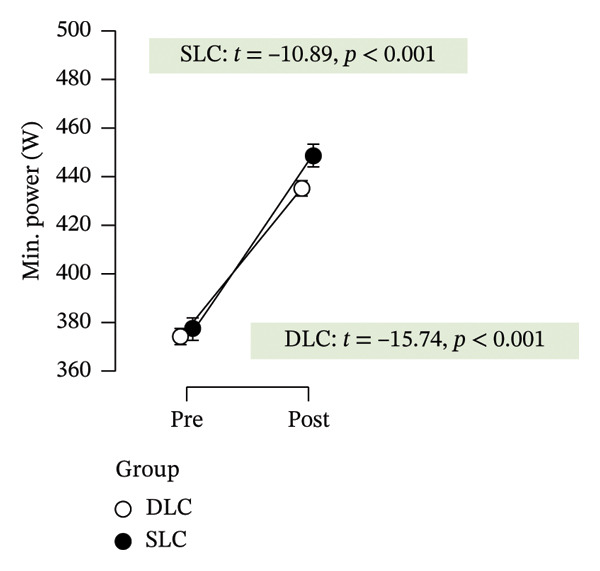
(b)

**Figure:**
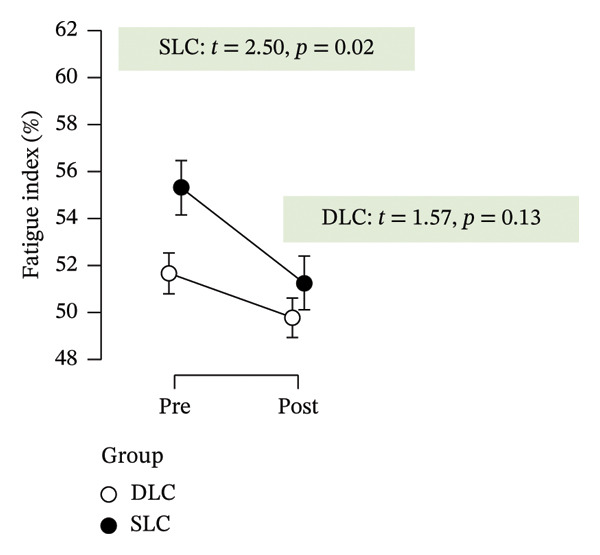
(c)

**Figure:**
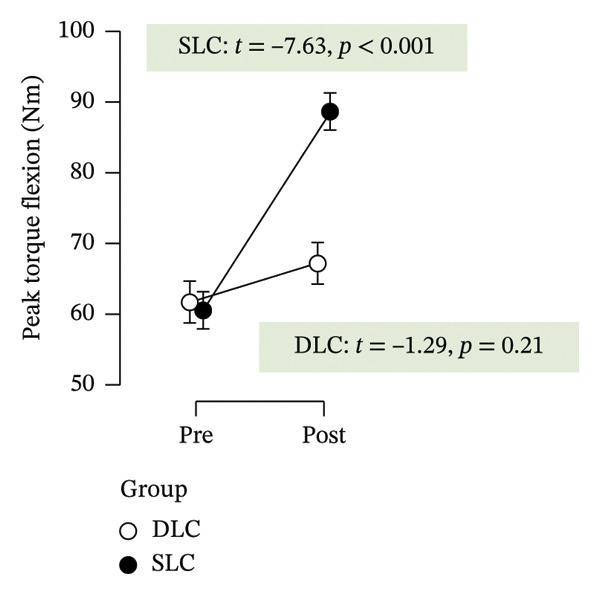
(d)

**Figure:**
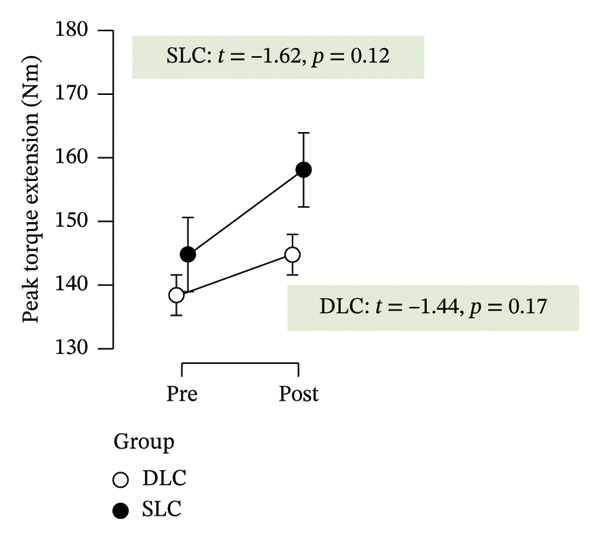
(e)

**Figure:**
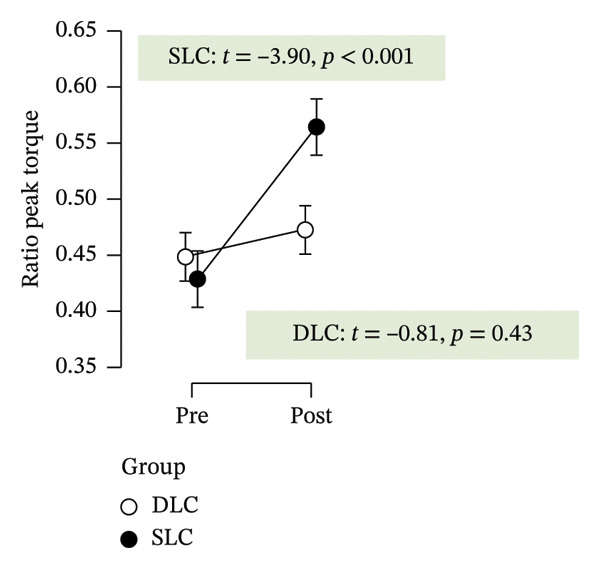
(f)

**Figure:**
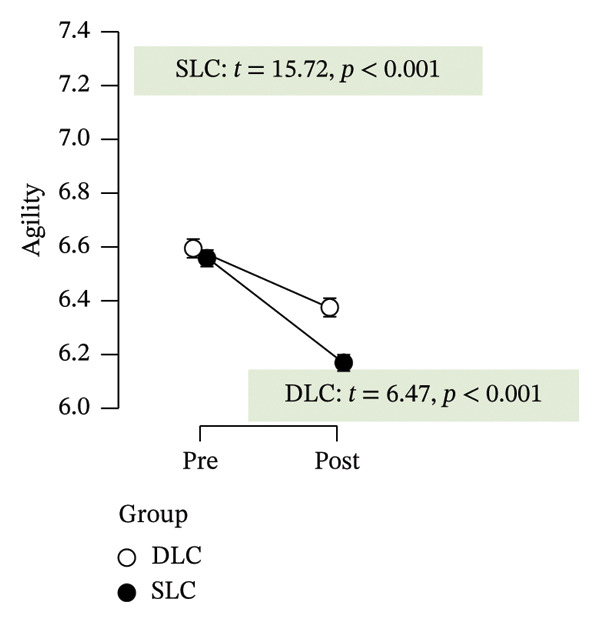
(g)

**Figure:**
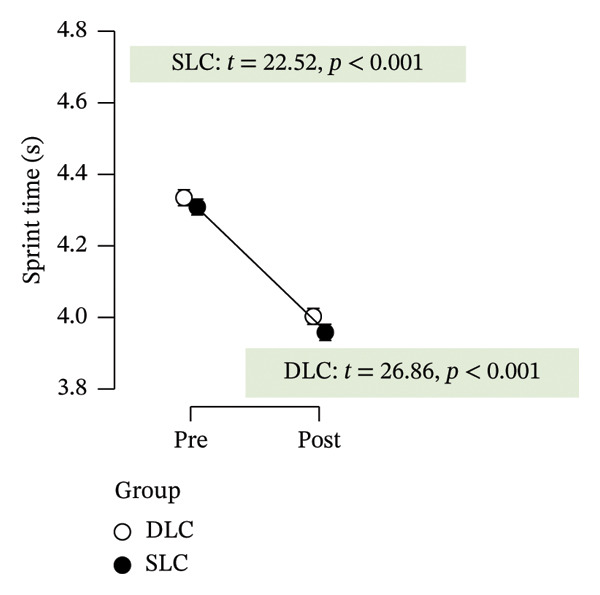
(h)

## 4. Discussion

The present study demonstrated significant improvements in peak power in both the SLC and DLC groups, with SLC yielding greater enhancements. This aligns with prior research indicating that unilateral cycling imposes greater neuromuscular demand, requiring the active limb to generate force independently without contralateral assistance [31]. SLC‐trained athletes exhibit lower exertion due to improved neuromuscular efficiency and motor unit recruitment [21, 32]. The increased motor unit recruitment and intermuscular coordination associated with SLC contribute to enhanced force production efficiency, which is particularly advantageous for football players performing explosive movements such as sprinting and rapid directional changes. Since football requires single‐leg propulsion for sprinting, cutting, and kicking, SLC may offer a sport‐specific overload stimulus [32]. Additionally, SLC has been shown to enhance muscle oxygenation and femoral blood flow, promoting increased power output during high‐intensity efforts [33]. The biomechanical benefits of unilateral cycling, including improved limb‐specific strength development, reinforce its potential as a valuable training tool for football athletes [15, 32].

Minimum power generated improved in both groups, with slightly greater gains in SLC, though the difference was not significant. This suggests that SLC may enhance power sustainability during repeated high‐intensity efforts, aligning with research on its benefits for mitochondrial efficiency, ATP resynthesis, and lactate tolerance [13, 21]. Fatigue resistance improved in the SLC group, while the DLC group showed no change. This suggests that SLC training may better enhance anaerobic endurance and delay performance decline. The greater fatigue resistance may result from improved mitochondrial efficiency and ATP resynthesis [13, 34]. Though metabolic markers were not measured, these adaptations likely aid power maintenance under anaerobic conditions. Unilateral cycling has been shown to enhance lactate metabolism and improve blood flow distribution, promoting efficient waste removal [12]. SLC’s higher metabolic demand may also enhance oxygen delivery and reduce performance decline [35]. These benefits are especially relevant in football, where late‐game fatigue affects execution and decision‐making, reinforcing SLC’s role in anaerobic conditioning.

The present study demonstrated improvements in knee flexor strength in both groups, with SLC exhibiting a more pronounced effect. This outcome may be attributed to the higher force demands placed on the hamstrings during unilateral cycling, leading to greater neuromuscular engagement and structural adaptations such as increased fascicle length and hypertrophy [36]. The improvements in knee flexor strength in SLC suggest a potential role in reducing hamstring injury risk, as stronger hamstrings contribute to improved joint stability and resilience [37]. However, further research is needed to confirm whether these strength adaptations directly translate to a lower injury incidence in football players. Previous research suggests that unilateral movements enhance stabilizing muscle, which contributes to the observed improvements in knee flexor strength [33]. The findings are supported by previous researches showing that athletes engaged in SLC interventions have shown superior hamstring strength and flexibility compared to those performing DLC [15]. However, the absence of surface electromyography (EMG) limits our ability to confirm muscle activation patterns during SLC versus DLC, particularly regarding the relative recruitment of hamstring and quadriceps muscles.

The study found minor improvements in quadriceps strength in both groups, with no substantial differences between SLC and DLC. This suggests that bilateral cycling may already provide an adequate stimulus for quadriceps development, and additional loading from unilateral cycling does not appear to offer further advantages. Prior research supports this notion, indicating that while SLC can induce muscle‐specific adaptations, overall quadriceps strength gains may require longer training durations or higher‐intensity resistance‐based interventions [34]. Quadriceps strength plays a crucial role in football‐related tasks such as kicking and tackling, emphasizing the need for targeted strength development [26]. Given the findings, future studies should investigate whether combining SLC with resistance training could yield greater quadriceps strength improvements. The relatively modest gains in the DLC group may reflect a more distributed neuromuscular load, where bilateral coordination reduces the relative intensity imposed on each limb, potentially limiting overload‐specific adaptations. While SLC has been suggested to improve neuromuscular coordination by engaging stabilizing muscles [12], the present findings indicate that these benefits may be more prominent in knee flexor strength rather than quadriceps development.

The 4‐week intervention is relatively short for improving strength; research has supported that measurable changes in performance can occur within similar timeframe and may contribute to long‐term outcomes also associated with neural and morphological changes in muscle function, supporting the physiological relevance of improved strength [38]. Moreover, previous findings have suggested that gains from the short‐term training can persist during the detraining periods indicating that these adaptations are not entirely transient and could form the foundation for further improvements with continued training [39, 40].

The present findings can be influenced by the external training exposure and limb‐specific loading. Participants continued their usual football training in the same volume and intensity throughout the intervention period, which was not monitored. Therefore, the improvement in the sprint performance and agility may reflect the combined effects of the football training‐ and cycling‐based conditioning rather than cycling intervention alone. However, random allocation of participants and standardized training sessions were conducted concurrently under the supervision of the same coaching staff, which minimizes the systematic between‐group differences in training exposure and to strengthen internal validity, as recommended for exercise intervention trials designed to evaluate causal effects of training stimuli [41].

The training intensity was regulated using perceived exertion and HR as an indicator of internal physiological load and cardiovascular stress. This method is used in previous studies for standardizing the relative exercise intensity and ensuring participant safety [14]. The previous research has demonstrated the weak positive correlation between the internal load measures and external load metrics such as HR [42]. Although HR does not directly quantify the external neuromuscular loading, it acts as a measure of physiological strain used to control intensity in sports science.

Isokinetic dynamometry was used to controlled and reliable assessment of quadriceps and hamstring strength and H/Q ratio. SLC has shown the significant improvement in the H/Q ratio, while only minor changes were observed in the DLC group. These variables are commonly used to evaluate the neuromuscular status and injury‐related risk in the football players. The findings suggest that SLC may be particularly beneficial for enhancing muscle balance, a critical factor in reducing the risk of anterior cruciate ligament (ACL) injuries and hamstring strains in football players [18, 37]. The greater hamstring activation required during SLC, combined with its unilateral nature, likely contributed to the superior H/Q ratio improvements observed. A balanced H/Q ratio has been linked to improved joint stability and lower injury susceptibility, emphasizing the importance of incorporating targeted training strategies to optimize this parameter [36]. The tested angular velocity (30°–180°/s) is the important aspect of the concentric strength and muscle balance, but it does not fully represent the eccentric high‐velocity contractions, which are characteristics of sprinting and cutting maneuvers. The findings of this study reflect the controlled neuromuscular adaptations rather than direct measures of the football‐specific movements. Prior studies have demonstrated that unilateral training, such as SLC, enhances neuromuscular coordination and stability during dynamic movements, reinforcing its potential as a preventive intervention for lower limb injuries in high‐intensity sports [33].

Both groups demonstrated significant improvements in agility and sprint performance, with SLC yielding slightly greater enhancements. These results align with prior research suggesting that SLC enhances neuromuscular coordination and peripheral adaptations, which are crucial for explosive movements in football [12, 43]. The unilateral nature of SLC promotes greater activation of stabilizing muscles, such as the hamstrings and hip abductors, which are essential for rapid directional changes during dribbling, tackling, and sprinting [35]. The superior agility and sprint gains in SLC may stem from enhanced neuromuscular synchronization and motor unit recruitment, both of which are critical for explosive football movements [32]. Given that football demands high‐speed, single‐leg propulsion actions, integrating SLC into training regimens could complement traditional methods by providing targeted overload for agility and sprint performance. Future research should explore hybrid training approaches that combine SLC with plyometric and resistance training to further optimize performance adaptations.

## 5. Limitations

This study has several limitations. First, due to the visible nature of the interventions, participant blinding was not feasible; however, outcome assessment blinding was maintained to minimize bias. Second, although validated, the use of handheld stopwatches for sprint and agility assessments may introduce minor timing inconsistencies. Third, the intervention duration was limited to 4 weeks, which may not capture long‐term neuromuscular adaptations or retention effects. Fourth, the isokinetic testing was conducted at fixed angular velocities (30°/s, 60°/s, and 180°/s), which may not fully replicate the high‐speed dynamic contractions observed in match play. The regular football training was not monitored for the workload during the intervention period, which might affect the results. Also, the mechanical workload for SLC and DLC groups was not matched; although the HR was used to regulate intensity, this does not fully capture the neuromuscular stress on lower limb musculature. The study has taken HR for monitoring the exercise intensity; although it is used as the measure of internal load, it does not directly quantify the neuromuscular loading. Additionally, the study focused exclusively on healthy male football players aged 18–26 competing at the district level, with relatively less training experience and known to influence responsiveness to conditioning program more as compared to subelite athletes. This limits generalizability to female athletes, elite or professional players or older age groups. Lastly, surface EMG was not employed, which could have provided insight into muscle activation patterns during the cycling protocols.

## 6. Conclusion

This study demonstrated that SLC is superior to DLC in enhancing neuromuscular conditioning in football players. SLC resulted in greater improvements in peak torque, particularly in knee flexor strength, along with a significant increase in the H/Q ratio, suggesting its potential for injury prevention. Additionally, anaerobic performance, including peak and minimum power, improved more in the SLC group, alongside a greater reduction in fatigue index, indicating enhanced metabolic efficiency and fatigue resistance. SLC effectively enhances lower limb strength and anaerobic performance, supporting its inclusion in football training programs.

Future studies should explore longer training durations and include follow‐up assessments to evaluate the sustainability of performance gains. The integration of EMG and biomechanical analysis is recommended to better understand the underlying neuromuscular mechanisms. Investigating the effects of SLC in different athletic populations—such as female players, youth athletes, or individuals undergoing rehabilitation—could enhance its applicability. Future studies should also examine whether similar neuromuscular adaptations occur in highly trained or professional football populations, where responsiveness to short‐term conditioning interventions may differ. Additionally, comparisons with other unilateral strength modalities (e.g., step‐ups, split squats) may help position SLC within broader conditioning frameworks.

## Funding

The authors declare that no funds, grants, or other support were received during the preparation of this manuscript.

## Conflicts of Interest

The authors declare no conflicts of interest.

## Data Availability

The datasets generated and analyzed during the current study are available from the corresponding author upon reasonable request.
